# Genome-wide association study of Fusarium ear rot disease in the U.S.A. maize inbred line collection

**DOI:** 10.1186/s12870-014-0372-6

**Published:** 2014-12-30

**Authors:** Charles T Zila, Funda Ogut, Maria C Romay, Candice A Gardner, Edward S Buckler, James B Holland

**Affiliations:** Department of Crop Science, North Carolina State University, Raleigh, North Carolina 27695 USA; Institute for Genomic Diversity, Biotechnology bldg., Cornell University, Ithaca, NY 14853 USA; U.S. Department of Agriculture—Agricultural Research Service, North Central Regional Plant Introduction Station, Ames, IA 50014 USA; U.S. Department of Agriculture—Agricultural Research Service, Plant, Soil, and Nutrition Research Unit and Department of Plant Breeding and Genetics, Bradfield Hall, Cornell University, Ithaca, NY 14853 USA; U.S. Department of Agriculture—Agricultural Research Service Plant Science Research Unit and Department of Crop Science, North Carolina State University, Raleigh, North Carolina 27695 USA

**Keywords:** Association analysis, Disease resistance, Genomic selection, Maize, Quantitative trait

## Abstract

**Background:**

Resistance to Fusarium ear rot of maize is a quantitative and complex trait. Marker-trait associations to date have had small additive effects and were inconsistent between previous studies, likely due to the combined effects of genetic heterogeneity and low power of detection of many small effect variants. The complexity of inheritance of resistance hinders the use marker-assisted selection for ear rot resistance.

**Results:**

We conducted a genome-wide association study (GWAS) for Fusarium ear rot resistance in a panel of 1687 diverse inbred lines from the USDA maize gene bank with 200,978 SNPs while controlling for background genetic relationships with a mixed model and identified seven single nucleotide polymorphisms (SNPs) in six genes associated with disease resistance in either the complete inbred panel (1687 lines with highly unbalanced phenotype data) or in a filtered inbred panel (734 lines with balanced phenotype data). Different sets of SNPs were detected as associated in the two different data sets. The alleles conferring greater disease resistance at all seven SNPs were rare overall (below 16%) and always higher in allele frequency in tropical maize than in temperate dent maize. Resampling analysis of the complete data set identified one robust SNP association detected as significant at a stringent *p*-value in 94% of data sets, each representing a random sample of 80% of the lines. All associated SNPs were in exons, but none of the genes had predicted functions with an obvious relationship to resistance to fungal infection.

**Conclusions:**

GWAS in a very diverse maize collection identified seven SNP variants each associated with between 1% and 3% of trait variation. Because of their small effects, the value of selection on these SNPs for improving resistance to Fusarium ear rot is limited. Selection to combine these resistance alleles combined with genomic selection to improve the polygenic background resistance might be fruitful. The genes associated with resistance provide candidate gene targets for further study of the biological pathways involved in this complex disease resistance.

**Electronic supplementary material:**

The online version of this article (doi:10.1186/s12870-014-0372-6) contains supplementary material, which is available to authorized users.

## Background

Fusarium ear rot disease of maize, caused by the fungus *Fusarium verticillioides* (Sacc) Nirenberg, is endemic to maize production systems in the United States and worldwide [1]. The fungus is present as a symptomless endophyte in most maize seed lots [2-4]; pathogenic colonization of developing maize kernels is common in the low rainfall high-humidity climates of the southern United States and lowland tropics [5]. Infection by *F. verticillioides* can result in decreased grain yield, reduced grain quality, and grain contamination by the mycotoxin fumonisin. Fumonisin is a suspected carcinogen and is associated with various diseases in livestock and humans [5-7]. In areas of the world where maize is a dietary staple and occurrence of Fusarium ear rot infection is high (such as sub-Saharan Africa), consumption of infected grain has been linked to esophageal cancer in adults and growth retardation in children [8-10].

The most effective method for controlling Fusarium ear rot infection and reducing fumonisin contamination is through the deployment of maize hybrids possessing genetic resistance. Resistance to the disease is under polygenic control, and no fully immune genotypes have been discovered [11-13]. Previous linkage-based and association mapping studies have shown that resistance quantitative trait loci (QTL) have relatively small effects and are not consistent between populations [14-17]. The complex nature of resistance has made it difficult for maize breeders to effectively incorporate novel resistance alleles into adapted breeding pools; as a result, most commercial maize hybrids have lower levels of resistance than desired [18]. Although the heritability of individual plot measures of resistance to Fusarium ear rot and fumonisin contamination is low, resistance on an entry mean-basis from replicated bi-parental and diversity panel studies is moderately to highly heritable [19-22]. Empirical studies demonstrate that phenotypic selection for improved ear rot resistance can be effective [21,23]. However, most novel sources of disease resistance are unadapted inbreds with poor agronomic performance that often come from tropical or other exotic germplasm pools [12,22].

Genome-wide association studies (GWAS) can be a powerful tool in the identification of specific allele variants that confer improved resistance to various diseases in maize. Utilizing a maize core diversity panel of 279 public inbred lines [24] and over 47,000 SNPs from the Illumina maize 50 k array [25], Zila et al. [22] identified three genes associated with improved resistance to Fusarium ear rot. However, the three loci associated with improved ear rot resistance all had small allelic effects (±1.1% on a percentage ear rot scale), and each individual locus was associated between 3 to 12% of the observed variation in line means after accounting for the additive polygenic background genetic variance captured by the genomic kinship matrix. The alleles conferring greater resistance at all three loci were at higher frequency in tropical maize than in temperate maize, suggesting that tropical germplasm is a good source of resistance alleles that might not be found easily in elite temperate maize. Therefore, further searches for new resistance alleles should target diverse, mostly tropical, maize germplasm.

The USDA-ARS North Central Regional Plant Introduction Station (NCRPIS) located in Ames, IA maintains a large and diverse collection of maize inbred lines that represents a century of public and private maize breeding efforts in the United States and from across the globe [26]. Within the last year, almost 680,000 genotype-by-sequencing (GBS; [27,28]) markers on 2,815 accessions from the NCRPIS collection have become available through the efforts of Romay et al. [26]. The availability of this large set of markers on the NCRPIS collection provides the opportunity for significantly expanding the sample of maize diversity and the marker density for GWAS studies in maize. The objectives of this study were to evaluate 1687 diverse inbred lines from the NCRPIS collection and a subset of their topcross hybrids for resistance to Fusarium ear rot across several years and to conduct genome-wide association studies of resistance to this important disease using a set of 200,978 GBS SNPs from Romay et al. [26].

## Results

### Line means and heritability

Significant (*P* < 0.001) genotypic variation for ear rot resistance was observed in both the inbred association panel and topcross experiments. Ear rot least squares means among 1687 entries of the inbred association panel ranged from 0.2% to 100% with a mean score of 38.5% (Table 1 and File S4 in Additional file 1). Least square means for topcross hybrids ranged from 2.5% to 84.8% with a mean score of 21.0%. Entry mean-basis heritability of ear rot resistance in the full inbred association panel was 0.21, while in the balanced subset of 734 entries all tested across three years it was 0.61. Heritability of topcross rot resistance averaged across testers (for the set of lines evaluated in combination with both testers) 0.63, while heritabilites of resistance within the B47 and PHZ51 topcross sets individually were 0.46 and 0.18, respectively. The genotypic correlations between inbred ear rot resistance and resistance in topcrosses to B47 and PHZ51 were 0.39 and 0.42, respectively. The genotypic correlation between performance of B47 topcrosses and PHZ51 topcrosses was 0.48. On an inbred *per se* basis, B47 had a mean ear rot score of 28.1%, whereas PHZ51 had a mean score of 58.7% (File S4 in Additional file 1).

**Table 1:** **Sample size (N), mean ear rot severity, genotypic variance component estimates** $\left({\hat{\boldsymbol{\sigma}}}_{\boldsymbol{G}}^{\mathbf{2}}\right)$ **, average prediction error variance** $\left({\boldsymbol{\sigma}}_{\boldsymbol{PPE}}^2\right)$ **and heritability** $\left({\hat{\boldsymbol{H}}}_{\boldsymbol{C}}\right)$ **estimates for Fusarium ear rot resistance in the full inbred association panel, filtered association panel, across the topcross experiment, and within the B47 and PHZ51 topcrosses, respectively**

	**N**	**Mean (%)** ^***a***^	${\left({\hat{\boldsymbol{\sigma}}}_{\boldsymbol{G}}^{\mathbf{2}}\right)}^{\boldsymbol{b}}$	${\left({\boldsymbol{\sigma}}_{\boldsymbol{PPE}}^{\mathbf{2}}\right)}^{\boldsymbol{c}}$	${\hat{\boldsymbol{H}}}_{\boldsymbol{C}}$
Full inbred panel	1687	38.5	0.15	0.24	0.21
Filtered inbred panel	734	33.0	0.18	0.14	0.61
Topcrosses	556	21.0	0.13	0.10	0.63
* B47*	243	23.1	0.15	0.16	0.46
* PHZ51*	313	19.4	0.06	0.10	0.18

^*a*^Mean ear rot severity is reported as the average of the entry least square means (back-transformed to the original 0-100% disease severity scale).

^*b*^Estimated genetic variance component from ASReml.

^*c*^Average prediction error variance among all pair-wise comparisons of entries from ASReml.

### Genome-wide association mapping of Fusarium ear rot resistance

**Figure 1:**
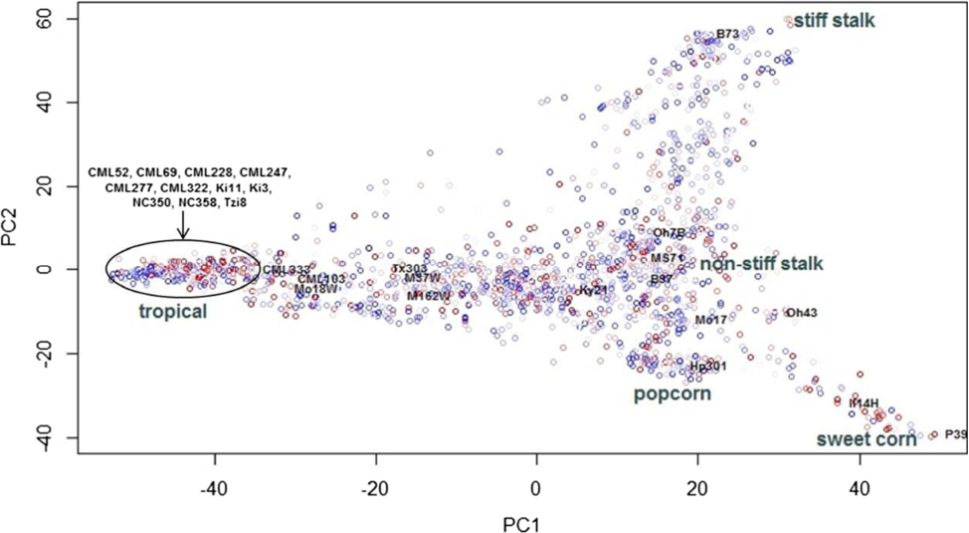
**Genetic relationships between the 1687 lines of the full inbred association panel visualized using a principal component analysis of the K matrix.** The horizontal and vertical axes are the first and second principal components, respectively. The color gradient from blue to red of the points represents the relative mean Fusarium ear rot score of each line (blue is most resistant and red is most susceptible). Five major recognized heterotic group clusters are labeled in large gray font, and the 26 nested association mapping (NAM) population founders and Mo17 are labeled in small black font for reference.

Background polygenic effects modeled by **K** accounted for 31% of the variation among entry means in the full inbred association panel analysis and 42% of the entry mean variation in the balanced subset inbred association panel (Table 2). Principal component decomposition of **K** revealed little association between mean rot scores in the inbred association panel and large-scale population structure (Figure 1). In the topcross analyses, **K** accounted for 31% of the variation among B47 topcross entry means and 39% of the variation among PHZ51 topcross entry means (Table 2).

**Table 2:** **Number of lines, number of groups and compression level of the full 2480 × 2480 kinship matrix, and proportion of total line mean variance explained by additive relationship matrix from the four mixed-linear model (MLM) analyses**

	**N** ^***a***^	**Groups** ^***b***^	**Compression** ^***c***^	${\left(\frac{{\hat{\boldsymbol{\upsigma}}}_{\mathbf{G}}^{\mathbf{2}}}{{\hat{\boldsymbol{\upsigma}}}_{\mathbf{G}}^{\mathbf{2}}+{\hat{\boldsymbol{\upsigma}}}^{\mathbf{2}}}\right)}^{\boldsymbol{d}}$
Full inbred panel	1687	2100	1.18	0.31
Filtered inbred panel	734	2000	1.24	0.42
B47 topcrosses	243	1760	1.41	0.31
PHZ51 topcrosses	313	1770	1.40	0.39

^*a*^Total number of entries included in the analysis.

^*b*^Number of groups determined by optimum compression (note that the complete kinship matrix for 2480 lines was used for all analyses).

^*c*^Compression level is the average number of individuals per group.

^*d*^Polygenic additive background genetic variance divided by total phenotypic variance. This ratio was estimated in GAPIT by fitting the kinship matrix (**K**) in the mixed linear model without any SNP marker effects.

**Figure 2:**
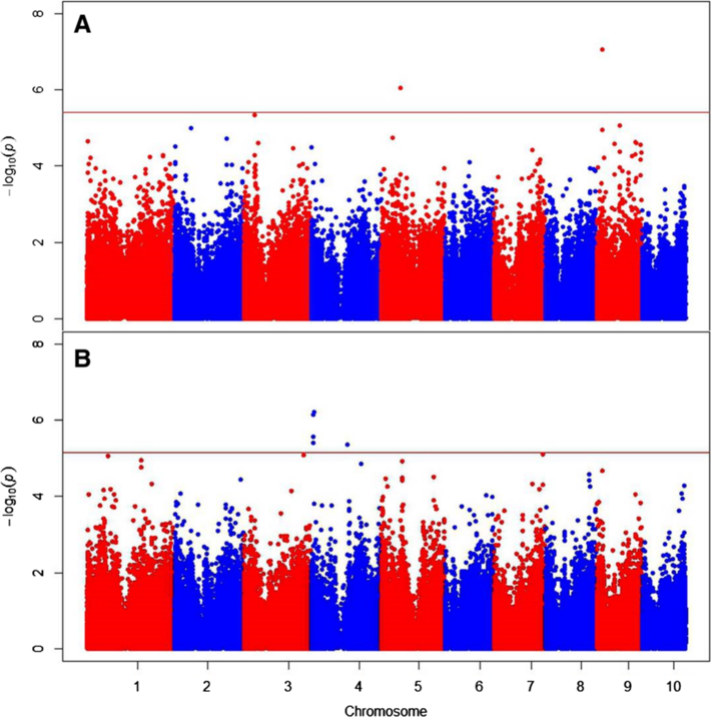
**Manhattan plots showing significant associations (points above the red FDR = 0.20 threshold lines) from the full inbred association panel (A) and filtered inbred association panel (B) GWAS analyses.** The vertical axis indicates –log_10_ of *P*-value scores, and the horizontal axis indicates chromosomes and physical position of SNPs.

From the analysis of the full inbred association panel, two SNPs (at bp 64,771,372 on chromosome 5 and at bp 19,532,465 on chromosome 9) were identified as significantly associated with ear rot resistance at a false discovery rate (FDR) of < 0.20 (Table 3; Figure 2). These two SNPs also had the highest RMIP values among SNPs across the 50 data subsamples; the chromosome 9 SNP had an association with ear rot with *p*-value < 10^−5^ in 47 of the 50 data subsamples (Table 3; Figure S1 in Additional file 1; File S6 in Additional file 1).

**Table 3:** **Chromosome locations (AGP v2 coordinates), allele effect estimates, genes containing SNP, and other summary statistics for the seven SNPs significantly associated with Fusarium ear rot resistance from the two inbred association panel analyses**

**Chromosome**	**SNP physical position (bp)**	***p*** **-value**	**FDR adjusted P-value**	**Minor allele frequency**	**Allele effect (%)** ^***a***^	**(** ***R*** ^**2**^ **)** ^***b***^	**Gene containing SNP**	**SNP effect**	**RMIP**
		*Full inbred panel (1689 lines) analysis*							
5	64,771,372	8.83 × 10^−7^	0.089	0.07	−0.170	1.3	GRMZM2G060659	mis-sense (A/T)	0.38
9	19,532,465	8.44 × 10^−8^	0.017	0.15	−0.134	1.5	GRMZM2G035665	mis-sense (V/A)	0.94
		*Filtered inbred panel (737 lines tested in three years) analysis*							
4	7,566,354	7.34 × 10^−7^	0.074	0.10	−0.230	2.9	GRMZM2G372364	intron variant	
4	7,618,125	2.67 × 10^−6^	0.175	0.10	−0.225	2.6	GRMZM2G012821	mis-sense (N/D)	
4	7,618,284	3.96 × 10^−6^	0.175	0.11	−0.205	2.5	GRMZM2G012821	mis-sense (D/N)	
4	9,353,851	6.14 × 10^−7^	0.074	0.07	−0.254	3.0	GRMZM2G419836	3′ UTR variant	
4	124,930,006	4.36 × 10^−6^	0.175	0.04	−0.271	2.5	GRMZM2G106752	mis-sense (L/S)	

^*a*^Allele effects are reported back-transformed to the original 0-100% disease severity scale. Effects are in reference to the minor allele.

^*b*^ *R* ^2^, proportion of total entry mean variance associated with a SNP after accounting for background polygenic variance.

**Figure 3:**
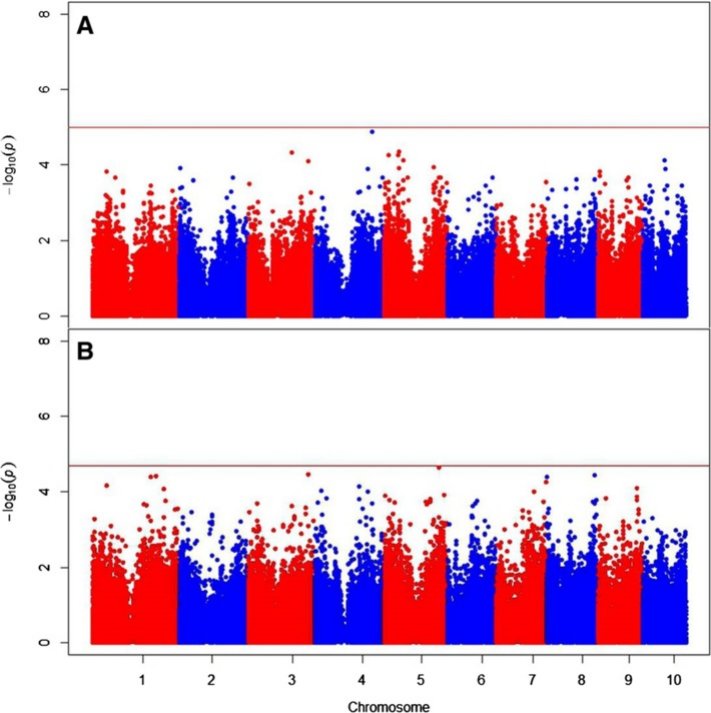
**Manhattan plots showing significant associations (points above the red FDR = 0.20 threshold lines) from the B47 topcross (A) and PHZ51 topcross (B) GWAS analyses.** The vertical axis indicates –log_10_ of *P*-value scores, and the horizontal axis indicates chromosomes and physical position of SNPs.

When the analysis was conducted on a filtered data set including only lines with data from all three years, a distinct set of five SNPs, all on chromosome 4, were identified as significantly associated with ear rot resistance (Table 3; Figure 2). No significant SNPs at FDR < 0.20 were identified from either the B47 topcross analysis or the PHZ51 topcross analysis (Figure 3), where the minimum raw *P*-values among SNP association tests were 1.3 × 10^−5^ and 2.3 × 10^−5^, respectively.

SNPs identified from either of the two inbred analyses explained relatively small proportions of the observed variance in entry means after accounting for the background polygenic effects (individual SNP *R*^2^ values ranged from 1.3% to 3.0%, Table 3), and each SNP also had a small allelic effect (−0.13% to −0.27% back-transformed to the original percentage ear rot scale). All significant associations had negative allelic effects, indicating that the minor allele was associated with lower ear rot (increased diseased resistance) at all loci.

The frequency of disease resistance alleles were estimated at the seven significantly associated SNPs in the same five major maize subpopulations analyzed by Zila et al. [22] – stiff stalk temperate (SS), non-stiff stalk temperate (NSS), tropical/subtropical (TS), popcorn (PC), and sweet corn (SC) [26]. Alleles associated with increased disease resistance at all seven SNP loci were significantly (*p* ≤ 1.7 × 10^−5^) overrepresented in the tropical and/or popcorn groups compared to the three other temperate groups (Table 4). Disease resistance alleles at all seven SNP loci were absent or nearly absent in the SS, NSS, and SC subpopulations. However, examination of the average of least squares means across lines sampled within a subpopulation showed no major difference in disease severity between the groups, largely agreeing with the principal component analysis of the **K** matrix (Table 4; Figure 1).

**Table 4:** **Allele frequencies of significantly associated SNPs in the five major maize subpopulations and** ***P*** **-value of Fisher’s exact test of the null hypothesis of equal allele frequencies across subpopulations**

		**Resistance allele frequency (%)** ^***a***^						**N** ^***b***^				
**Chromo-some**	**SNP physical position (bp)**	**SS** ^***c***^	**NSS**	**TS**	**PC**	**SC**	***P-*** **value**	**SS**	**NSS**	**TS**	**PC**	**SC**
4	7,566,354	1.2	0.0	32.0	60.4	0.0	<2.2 × 10^−16^	164	171	222	48	51
4	7,618,125	0.6	0.0	30.7	47.9	0.0	<2.2 × 10^−16^	164	171	215	48	52
4	7,618,284	0.6	0.0	36.5	66.0	0.0	<2.2 × 10^−16^	159	168	211	47	51
4	9,353,851	0.6	0.6	31.9	0.0	0.0	<2.2 × 10^−16^	161	168	213	61	50
4	124,930,006	0.0	1.8	8.8	3.3	0.0	1.7 × 10^−5^	162	166	238	60	51
5	64,771,372	0.0	4.7	8.1	14.8	0.0	3.2 × 10^−6^	164	170	246	61	51
9	19,532,465	2.5	7.2	26.6	26.7	2.0	4.9 × 10^−15^	158	167	241	60	51
	Ear rot mean (%)^*d*^	39.6	39.6	43.0	41.3	61.1						

^*a*^At all SNP loci the minor allele is associated with increased disease resistance.

^*b*^N, total number of lines within each subpopulation with marker calls at a particular SNP locus.

^*c*^SS, stiff stalk; NSS, non-stiff stalk; TS, tropical/subtropical; PC, popcorn; SC, sweet corn.

^*d*^Overall phenotypic ear rot means are the average of least squares means across members of each subpopulation.

### Genes colocalized with associated SNPs

**Figure 4:**
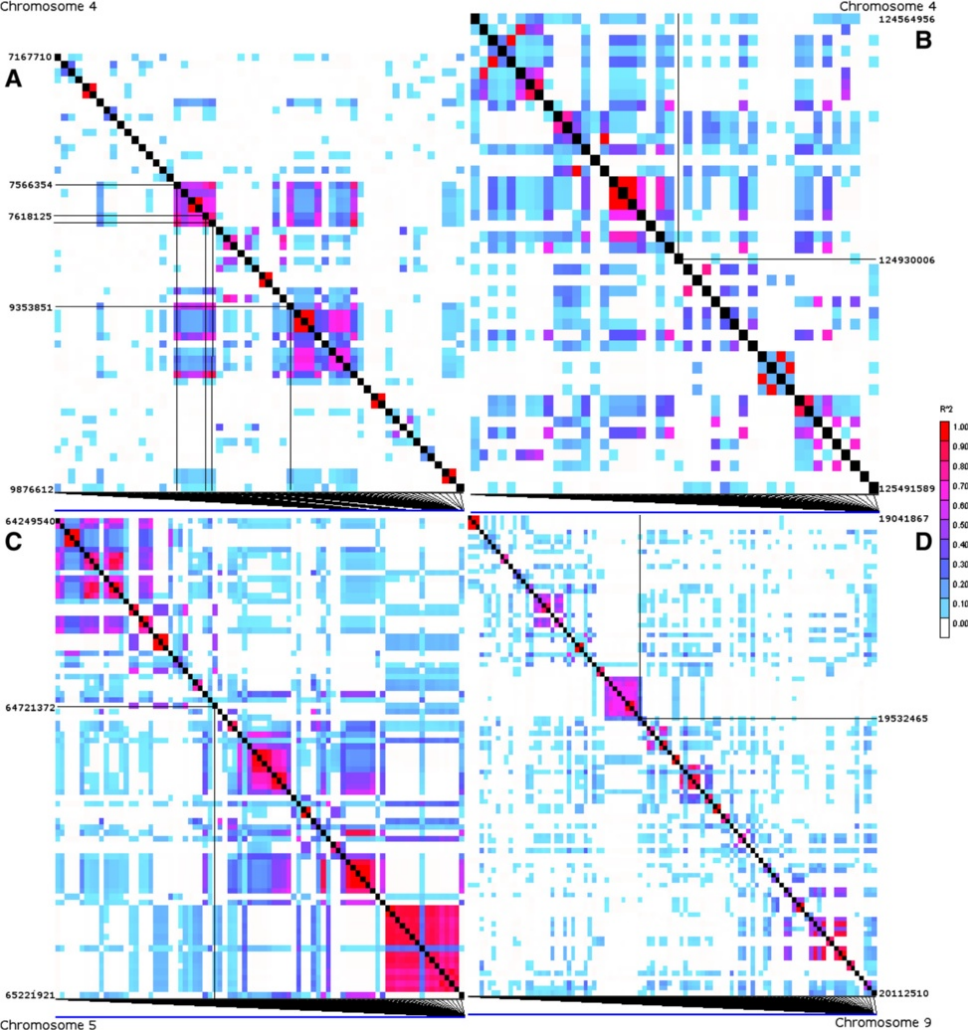
**LD heatmaps showing LD measure (** ***r*** ^**2**^ **) calculated for each pair-wise combination of SNPs in an approximately ±0.5 Mbp region surrounding each SNP significantly associated with ear rot resistance in the two inbred association panel analyses. (A)** LD around the four SNPs chromosome 4 SNPs located in the 7.6 Mbp to 9.4 Mbp interval. **(B)** LD around chromosome 4 SNP at physical position 124.9 Mbp. **(C)** LD around chromosome 5 SNP. **(D)** LD around chromosome 9 SNP. The significant SNP(s) on each chromosome is highlighted by the perpendicular black lines within each heatmap. Colors indicate the magnitude of each pair-wise *r* ^2^ measure (*r* ^2^ = 1 is red to *r* ^2^ = 0 is white).

**Figure 5:**
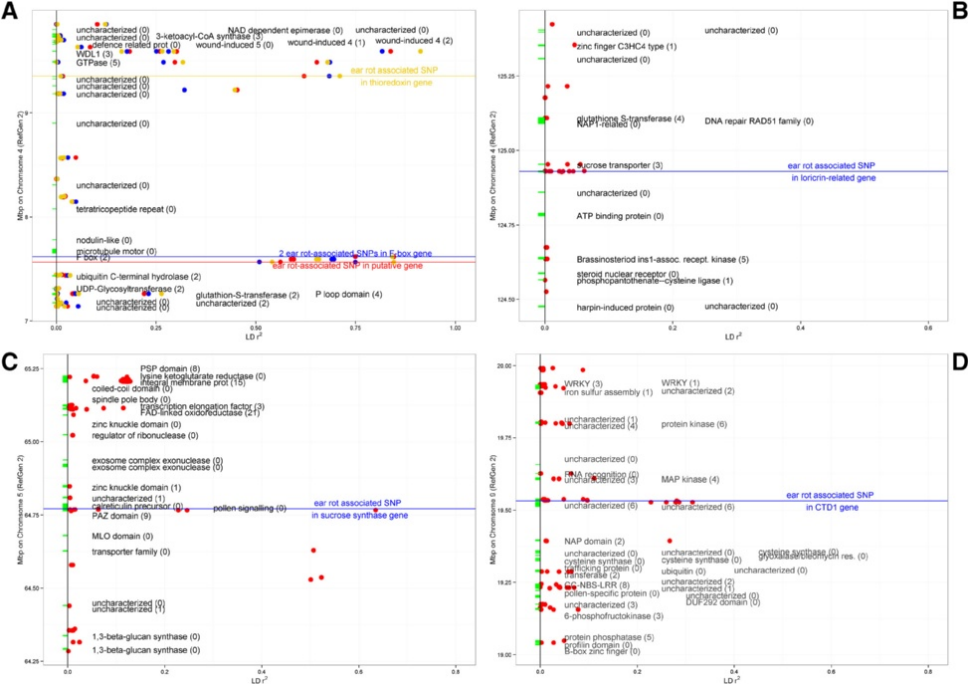
**Local gene annotations, SNP density, and LD** ***r*** ^**2**^ **between each SNP within 0.5 Mbp of a SNP association.** Positions of genes in the filtered gene set are shown as green boxes on Y-axis, brief annotations of the genes are shown along with the number of SNPs scored in the gene in parenthesis. SNPs are colored circles, their position on X-axis represent their LD *r* ^2^ with respect to the SNP reported as significantly associated with Fusarium ear rot. Note that the X-axis limits vary. The positions of significantly associated SNPs are indicated with horizontal lines. **(A)** Four significant SNPs located in the 7.6 Mbp to 9.4 Mbp interval on chromosome 4 displayed with different colors. The color of circles indicates the significant SNP to which the pairwise LD estimate refers. Two SNPs are located in an F-box gene so closely that their positions and LD values with other SNPs cannot be distinguished at this scale; their LD estimates are shown in blue. **(B)** A 1-Mbp region around a significantly associated SNP at 124,930,006 bp on chromosome 4. **(C)** A 1-Mbp region around a significantly associated SNP at 64,771,372 bp on chromosome 5. **(D)** A 1-Mbp region around a significantly associated SNP at 19,532,465 bp on chromosome 9.

To gauge the resolution of associations, we inspected the local LD structure around the significant associations (Figures 4 and 5). Romay et al. [26] summarized the genome-wide LD characteristics of this panel, noting that LD tends to decay rapidly to below *r*^2^ = 0.2 within 1 kb, but that there is substantial variation around this average value among genome regions and germplasm groups. The regions around our associations on Chromosome 4 near 125 Mb and on Chromosome 9 exhibit the typical rapid decay of LD observed in diverse maize. LD was slightly more extensive around the Chromosome 5 association, with a few SNPs about 200 kb away from the significant association having *r*^2^ of about 0.5 with the associated SNP. Finally, the region on Chromosome 4 between 7.5 and 9.5 Mb had the most extensive LD, with SNPs separated by almost 2 Mb still having high LD, although much of the region between the ends of this section had much lower LD. Romay et al. [26] observed that Chromosome 4 has particularly high LD. The high LD region reported here is coincident with the interval containing the *gametophyte factor 1* (*Ga1*) locus [29], which is under selection in the popcorn subgroup and may also be more widespread in tropical maize due to selfish gene evolution [30]. These selection effects associated with *Ga1* may be involved in maintaining LD in the region.

Genes containing SNPs significantly associated with ear rot resistance were characterized using the filtered predicted gene set from the annotated B73 reference genome [31] (Additional file 1: File S7). All seven SNPs identified across both inbred association panel analyses were within predicted genes on the maize physical map, five of the seven localized to exons (all coding for nonsynonymous mis-sense variations), one to the 3′ untranslated region, and one to an intron (Table 3). The disease associated SNP on chromosome 5 was in a sucrose synthase gene (GRMZM2G060659) located in an LD block extending approximately 0.2 Mbp on chromosome 5 (Figures 4C and 5C). Examination of the lines carrying the minor allele at this locus revealed no relationship between population structure due to kernel type (namely the sweet corn and popcorn groups) and presence of the minor allele. The associated SNP on chromosome 9 was in a DNA replication factor *CDT1*-like gene (GRMZM2G035665) located at the end of a 0.1 Mbp LD block on chromosome 9 (Figures 4D and 5D). All five SNPs identified in the balanced subset of the inbred association panel analysis were located on chromosome 4 (Figures 4A, B and 5A, B). Four of those SNPs were located in a 1.8 Mbp region between physical positions 7,566,354 bp and 9,353,851 bp, representing a region of high linkage disequilibrium covering a genetic distance of less than 1 cM (Liu *et al.* 2009) (Figure 4A). The four SNPs in this region were all in high LD relationships with each other (*r*^2^ from 0.62 to 0.84; Figure 5A). Two of the SNPs in this region localized to an exon of an F-box domain gene, one localized to a thioredoxin gene, and the last localized to a gene of no known function (GRMZM2G012821, GRMZM2G419836, and GRMZM2G372364, respectively). The fifth SNP identified on chromosome 4 located at position 124,930,006 bp localized to an exon of a loricrin-related gene (GRMZM2G106752).

## Discussion

### Heritability and genotypic correlation between experiments

The removal of lines that were not tested in all three years (consisting mostly of 953 unreplicated inbred lines that were present only in the 2010 NCPRIS collection experiment) substantially improved the entry mean-basis heritability. (${\hat{H}}_C=0.21$ in full data set versus ${\hat{H}}_C=0.61$ in filtered data set). This large difference in heritability provided justification for conducting separate GWAS on the complete and filtered inbred association panel data sets. Improved heritability of the mean values from the filtered panel will contribute to increased power of GWAS [32], but this is balanced by the loss of diversity and reduced allele replication in the subset compared to the complete set of inbreds. Analyses on the full versus filtered inbred data sets identified different genomic regions significantly associated with Fusarium ear rot resistance (Table 3). These differing results presumably reflect the tradeoffs between higher heritability and larger sample size that affect GWAS power.

Although the heritability estimate for ear rot resistance averaged across testers in the topcross experiment $\left({\hat{H}}_C=0.63\right)$ was comparable to that of the filtered inbred data set, no SNPs were identified as being significantly associated with ear rot resistance in either the B47 or PHZ51 topcross data sets. Estimates of genetic variance in the heritability calculations revealed reduced genetic variance in the topcross experiment compared to the inbred experiments (Table 1). Smaller genotypic sample size of the topcross experiment also contributes to reduced power of detection of SNP associations. In addition, genotypic correlations between inbred *per se* resistance and hybrid performance in the two sets of topcrosses were moderately low (*r*_g_ ≤ 0.42).

### Association mapping

Two SNPs significantly associated with ear rot resistance, located on chromosomes 5 and 9, respectively, were identified in the full inbred association panel analysis, and five additional SNPs (representing two different LD blocks) were identified on chromosome 4 in the filtered inbred panel analysis (Table 3). Although all SNPs localized to genic regions, no obvious relationship exists between the predicted functions of these genes and Fusarium ear rot resistance; however the currently limited understanding of pathways contributing to resistance restricts our ability to predict what genes might be involved in resistance to this complex disease.

These SNP associations are different than those previously reported by Zila et al. [22] based on analysis a subset of 267 lines with a smaller and largely distinct set of SNPs. The closest pair of associations between the two studies were the SNPs on chromosome 5, which localized to the same genomic bin; however, they are 34 Mbp distant from each other physically, and 14.4 cM apart genetically [33]. The differences between the results presented here and those reported by Zila et al. [22] may be due to sample size and sampling of alleles and also due to differences in the SNPs tested for association. None of the three SNPs reported as associated with ear rot resistance by Zila et al. [22], located on chromosomes 1 (63,540,590 bp), 5 (30,997,717 bp), and 9 (151,295,233 bp), were present in the filtered GBS Romay et al. [26] marker set, and thus we had no potential to detect them in this study. The nearest neighboring filtered GBS SNP to each of the three SNPs reported by Zila et al. [22] were located 82 bp (raw *p* = 0.44), 2902 bp (raw *p* = 0.74), and 299 bp away (raw *p* = 0.11), respectively. However, the chromosome 9 SNP from the Zila et al. [22] study located was present in the original unfiltered Romay et al. [26] marker set, but a follow-up analysis of this single marker in GAPIT using the full inbred panel found it insignificant (raw *P* = 0.78). Finally, only the three SNPs in the LD block from 7.5 – 9.2 Mb on Chromosome 4 in this analysis colocalized with any QTL intervals identified in two biparental families by Robertson-Hoyt et al. [15]. QTL positions for Fusarium ear rot are not consistent among biparental families [15,16], but this one QTL region on Chromosome 4 was exceptional in being identified by linkage in two families by Robertson-Hoyt et al. [15] and by association in this study.

The variability in SNP association results among different germplasm samples may be due in part to the relatively small effect sizes of the potentially many underlying causal variations, coupled with low frequency of many variants and rapid decay of LD in diverse maize germplasm. This could result in a situation where even SNPs physically close to a causal variant are not likely to be associated with enough phenotypic effect to permit their robust and reliable detection through association analysis in diverse populations. The high frequency of detection of the chromosome 9 SNP (in nearly all random subsamples of 80% of the full data set; Figure S1 in Additional file 1; File S6 in Additional file 1), and the consistency of its effect even in the filtered subsample (where although it did not pass the FDR threshold of 0.2, its raw *p*-value was 2.15 × 10^−5^, Additional file 1: Table S1), suggest that its association in very diverse maize is reliable.

The five SNPs on chromosome 4 that were detected in the filtered but not the complete inbred panel had substantially higher allele effect estimates in the filtered panel, but similar allele frequencies across panels (Table S1 in Additional file 1). The difference in these results may be due to a reduction in the influence of many line means with only a single environment observation associated with a lower heritability in the full inbred line panel, and possibly greater precision of the resulting allele effect estimates. In contrast, the two SNPs detected in the full panel had consistent allele frequencies and effect estimates across the two analyses, but simply did not have sufficient statistical significance to stand out among the hundreds of thousands of tests performed (Table S1 in Additional file 1).

Although this study used four times the number of SNP markers (200 k versus 47 k) and an association panel almost six times as large as those used by Zila et al. [22], the number of genic regions identified as significantly associated with ear rot was about the same for the two studies (four and three, respectively). Furthermore, the proportion of phenotypic variance among entry means explained on average by the **K** matrix across the two inbred analyses and two topcross analyses was similar to results reported by Zila et al. [22]. These results suggest that the genetic architecture of resistance to Fusarium ear rot is highly polygenic, with substantial genetic variability generated by a large number of effective variants, each with individually small effects. Even with increased marker coverage and a larger association panel, the results of this study highlight the limitations of GWAS to precisely identify allele variants with small effects on complex traits.

Marker coverage in this study is still insufficient to provide SNPs in high LD with all segregating sequence variants; Romay et al. [26] suggested that more than 700,000 SNPs would be required to tag almost all variant regions in diverse maize. Examination of the annotated genes around the significant associations reveals a number of genes nearby that contain no SNPs in our data set (Figure 5; Additional file 1: File S7), suggesting that we are likely to miss some true associations. Thus, it is possible that a further increase in marker density might reveal more SNP associations and possibly some genetic variants with larger effects. However, if the genetic architecture really is highly polygenic, then the benefit of increasing marker density on increasing the likelihood of tagging additional causal variations by LD association is likely to offset by the increasingly stringent significance thresholds imposed by the larger number of association tests conducted. The additional benefit of adding markers is also somewhat limited if most of the markers have low minor allele frequency (MAF), as is the case for the GBS markers used here [26]. The SNP associations detected in this study had minor allele frequencies ranging from 0.04 to 0.15 (missing phenotypic observations caused some markers to have MAF < 0.05 in the GWAS), compared to minor allele frequencies below 0.05 for more than half of the complete GBS marker set. Besides having low power of detection just due to reduced allele replication, rare alleles tend to be highly associated with population structure since they are usually limited to a single subpopulation, thereby further reducing their potential for trait association following correction for population structure. In this study, we removed SNPs with MAF < 0.05 to ensure reliable associations based on sufficient replication across lines. If rare alleles are a major component of the genetic architecture, however, we may have missed many important associations by dropping SNPs with low allele frequencies that would represent the best possible associations with rare functional alleles. Further studies would be required to better understand the compromises between improving reliability of results by removing rare SNPs versus potentially missing important but rare functional variants.

No significant SNPs were identified in either topcross analysis, and examination of the empirical distribution of *P*-values from the four analyses revealed a tendency towards higher *P*-values in the two topcross analyses compared to the two inbred panel analyses (Figures S2, S3, S4, and S5 in Additional file 1). Heterosis plays a significant part in Fusarium ear rot resistance, reducing both genetic variance and the mean level of disease in F_1_ hybrids compared to inbred parents [34], which can reduce the ability to discriminate levels of disease resistance in topcross hybrids. Further, within a set of hybrids created from crosses to a common tester, each topcross hybrid has an equal contribution of half of its alleles at all loci from the common tester, which also reduces genetic variation among the hybrids. The reduction of genetic variance, along with the smaller sample sizes, reduced the power of detection in hybrids relative to inbreds.

### Candidate genes for Fusarium ear rot resistance

Genetic and biochemical pathways leading to resistance to Fusarium ear rot are entirely unknown. Therefore, GWAS provides a forward genetics approach to screen efficiently many thousands of genes for association with the phenotype without requiring assumptions about what gene functions might be involved in resistance. The SNP associations reported here may help suggest and prioritize candidate genes for resistance to Fusarium ear rot, although we emphasize that associations between genetic variants and phenotypes do not imply that either the SNP is a functional variant or even that the gene containing the SNP is causally involved in resistance. Independent studies, particularly focusing on the biology of the gene functions in relation to infection of maize seeds or other plant tissues, will be required to determine if any of the genes identified here have a role in Fusarium ear rot resistance. Conversely, we expect that GWAS was unable to identify some true functional variants because of the combined effects of small effect size, allele frequency, limited LD in maize, and insufficient SNP density.

The genes containing significant SNP associations in this study include a thioredoxin gene, an F-box gene, a loricrin gene, a sucrose synthase, a CTD1 gene, and a gene of unknown function. The common theme among the likely functions of these genes is that they are very generally important for a variety of cellular processes. The thioredoxin protein family is involved in redox signaling for nearly every plant cellular process [35]; F-box genes are one of the most abundant gene superfamilies in plants and their protein products are involved in uniquitination and degradation [36]; loricrin is likely to be involved in cell membrane function, sucrose synthase is a key enzyme in plant metabolism, and CTD1 is involved in DNA replication. Because of the generality and importance of these gene classes, variation in their function is expected to affect a variety of cellular mechanisms, complicating their possible functional relationship to Fusarium ear rot resistance. Thus, our ignorance of the pathways to resistance to Fusarium suggests that the gene containing a SNP association but no known function should have similar priority for further research as the other candidate genes.

In addition to the genes containing the associated SNPs, there are some cases where LD appears to be sufficiently extensive as to suggest other genes in the region may be important. Around the associations reported between 7.5 and 9.4 Mb on Chromosome 4, for example, it is clear that SNPs in a number of genes across this nearly 2 Mb region are in high LD and will share the association signal with the functional variant in this region. There is a nearby cluster of defense-related and wound-induced proteins around 9.6 to 9.7 Mb that might be considered as putative candidates for further research. Two of those genes had no SNPs in our data set, so we cannot test their associations directly with these data. A few other genes very close to some of significant association also lacked SNPs for testing (Figure 5; Additonal file 1: File S7), and these could not be ruled out as potential candidates. Outside of the region on the short of Chromosome 4, however, the LD decay appears so rapid that it seems unlikely that the SNP associations are more than a few kb from a functional variant. Finally, we also note that there are some larger intergenic regions that lack SNPs (Figure 5), and some sequence variation in these regions may impact gene regulation important to ear rot resistance, but we are likely to miss many such variants in our GWAS scan.

## Conclusions

Zila et al. [22] suggested that GWAS could be a useful tool for identifying specific disease resistance allele variants in unadapted maize germplasm, thereby allowing maize breeders to more effectively introgress specific allele variants into adapted germplasm. However, the small effects of resistance loci identified in this study and Zila et al. [22] suggest that introgressing a few specific resistance loci may not have a large overall impact on resistance levels within temperate breeding populations. Directly targeting low frequency SNP alleles, particularly when they are harbored in unadapted subpopulations like the tropical and popcorn populations identified both here and by Zila et al. [22], combined with genomic selection for the polygenic background for both the target trait and general adaptation traits (which will favor selection of individuals with higher proportions of adapted alleles), however, may be a useful compromise to leverage the benefits of both approaches to prediction and selection, although the effectiveness of such schemes will depend in part on the targeted SNPs having a consistent association with a significant proportion of genotypic variation [37].

## Methods

### Germplasm and experimental design

In 2010, the NCRPIS collection of inbred lines [26] was evaluated for disease resistance at the Central Crops Research Station in Clayton, NC. The 2010 field experiment consisted of 2572 inbred line entries and was arranged in an augmented single replicate design. Experimental entries were divided into 18 sets of differing sizes based on maturity and field assignment, and sets were then randomly subdivided into incomplete blocks (where the maximum block size across sets was 23 plots). Each block within each set was augmented with a B73 check plot in a random position, and five other checks of varying maturities (IL14H, Ki11, P39, SA24, and Tx303) were included once per set in a random position.

In 2011 and 2012, a novel association mapping panel consisting of 771 diverse inbred line entries was evaluated for disease resistance in Clayton, NC. Based on phenotypic information from the 2010 field experiment, a subset of 681 inbred lines from the NCRPIS collection representing a range of both pedigrees and disease severity scores was chosen for the panel. An additional 90 lines, mostly modern public lines available from North Carolina State University as well as a few lines developed by private industry with recently expired Plant Variety Protection Act (exPVPA) coverage that had become available through the NCRPIS in the spring of 2011 were included. The complete panel of 771 entries was divided into eight sets based on maturity and replicated across the two years using an augmented design. Within years, sets were randomized within the field, and each set was blocked using an *α*-lattice design [38]. Similar to the NCRPIS evaluation, each block was augmented by a randomly assigned B73 check plot, and five other checks representing a range of maturities and disease reactions (GE440, NC358, 794, B47, and Tx303) were included once per set.

Topcross F_1_ hybrids representing a subset of inbred lines from the 2011–2012 association panel were also evaluated in Clayton, NC in 2011 and 2012. Due to seed availability, topcross seed was limited to a sample of 405 inbred lines from the total 771 entries of the association panel. F_1_ hybrid seed was generated by crossing inbred lines to either the stiff stalk exPVPA inbred tester PHB47 or the non-stiff stalk exPVPA inbred tester PHZ51 (or both). Overall, 92 lines were crossed only to B47, 162 lines were crossed only to PHZ51, and 151 lines were crossed to both testers, resulting in a total of 556 F_1_ hybrid entries in the topcross panel. In the 2011 and 2012 field experiments, topcross entries were classified by tester and maturity (early or late, for a total of four tester × maturity combinations), and each tester × maturity combination was randomly subdivided into three groups. One random group of each tester × maturity combination was assigned to a set, for total of three sets (with four groups per set). Similar to the inbred association panel, sets were randomized within the field in each year, groups were randomized within set, and each group was then subdivided into incomplete blocks, but the topcross hybrids were grown in different field blocks than the inbreds. Each block was augmented with a B73 × PHZ51 topcross check plot in a random position, and two other hybrids that exhibited relatively good resistance to Fusarium ear rot in previous experiments (Pioneer 31G66 and NC478 × GE440) were included once per group. Lastly, one additional check plot of P39 × PHZ51 or CML52 × PHZ51 was included once per group depending on maturity (early or late, respectively).

### Inoculation and phenotyping methods

The 2010 NCPRIS collection experiment and the 2011/2012 inbred association panel experiments were inoculated with local toxigenic *Fusarium verticillioides* isolates using the toothpick method [12,22]. Approximately one week after flowering, a toothpick containing dried *F. verticillioides* conidia was inserted near the base of the primary ear of five plants in each plot. At maturity, inoculated ears were harvested and visually scored for Fusarium ear rot symptoms. Scores were assigned to each ear in increments of 5% from 0% to 100% diseased based on the percentage of the ear displaying disease symptoms [19].

Topcross hybrid experiments in 2011 and 2012 were inoculated with a suspension of *F. verticillioides* conidia using the method described by Robertson et al. [19]. Approximately one week after flowering, 5 mL of a liquid suspension containing 2 × 10^6^ conidia mL^−1^ was injected into the silk channel of the primary ear of five plants in each plot. One week following the first inoculation, 5 mL of the conidia suspension was injected near the base of the primary ear of the same plants inoculated in the first week. At maturity, inoculated ears were harvested and visually scored using the same protocol as the inbred disease experiments. Raw data from both the inbred and topcross experiments are provided in supplemental datasets File S1 and File S2 in Additional file 1, respectively.

### Genotypic data

The genotypic data used in this study consisted of 200,978 SNPs filtered from the GBS markers developed by Romay et al. [26]. The original set of markers consisted of 681,257 SNPs generated by the approach described by Elshire et al. [27] and Glaubitz et al. [28] with missing data imputed using the haplotype-based imputation method described by Romay et al. [26]. SNP data are available at http://panzea.org/db/gateway?file_id=Romay_etal_2013_imputed_geno_data. In addition, the Romay et al. [26] marker set was augmented with GBS data for the ninety inbred lines in the 2011/2012 association panel that were not present in the NCPRIS collection in 2010. GBS data for the aforementioned lines were obtained through the Institute for Genomic Diversity at Cornell Unversity, Ithaca, NY (http://www.igd.cornell.edu). Even after haplotype-based imputation, some missing genotypes exist because the imputation method of Romay et al. [26] does not impute missing data when the observed scores within a test haplotype window do not sufficiently match the reference haplotype set. Therefore, the augmented SNP marker set was then filtered to include only those markers that had less than 20% missing data (after haplotype-based imputation) and a minor allele frequency (MAF) greater than 5%. Duplicate samples present in the Romay et al. [26] data set were also removed from the augmented data set; after this filtering step, genotypic data were available for a total of 2480 inbred lines from across all years combined. The final genotypic data set used in the GWAS analyses is provided in supplemental dataset File S3 in Additional file 1.

### Statistical analyses

#### Estimation of least square means

Fusarium ear rot data from the 2010 NCPRIS collection experiment and the 2011/2012 inbred association panel experiments were first analyzed separately to determine the best fitting spatial model within each year, and then the best models within each year were combined together to form a single multi-environment trial analysis. Within each year, a model was first fit with a fixed entry effect, fixed first, second, third, and fourth order polynomial trend effects in both the row and column directions [39], and flowering time as a fixed linear covariate. Only those fixed trend effects significant at *P* < 0.01 were chosen to remain in the model, and flowering time was also dropped from the model if it was not significant at *P* < 0.05. Once significant fixed effects were selected, random effects were chosen using Akaike’s Information Criterion [40] to compare four different models within each year: a model fitting only the significant fixed effects; a model fitting significant fixed effects and random set and block within set effects; a model fitting fixed effects and an anisotropic correlated error structure [39]; and a model fitting fixed effects, random set and block within set effects, and an anisotropic correlated error structure. All models were weighted by the number of ears scored within each plot, and a natural logarithmic transformation of raw ear rot scores was used in all analyses due to an association between the magnitude of predicted ear rot values and residuals. All analyses were performed using ASReml version 3 software [41].

Once the best model within each year was selected, a single multi-environment trial analysis was conducted by nesting the various best spatial models within year. Fixed effects from the individual year analyses were checked again for significance in the combined model, and those which became insignificant in the combined model were dropped. The combined model had the form:

$$ {Y}_{ijkl}=\mu +YEA{R}_i+ SET{(YEAR)}_{ij}+ BLOCK{\left( SET\times YEAR\right)}_{ijk}+{x}_{r- ijkl}{\beta}_{row}+{x}_{c- ijkl}^2{\beta}_{col}+LIN{E}_l+ LINE\times YEA{R}_{il}+{\varepsilon}_{ijkl}. $$

The effects in this model were a fixed entry (line) effect (LINE_l_), random year (YEAR_i_) and line × year effects, a heterogeneous error variance structure within each year ε_ijkl_ (with unique variances in each year), and the various spatial effects nested within their respective years: a random set effect in 2010 (SET(YEAR)_ij_), a random block within set effect in 2010 (BLOCK(SET × YEAR)_ijk_), a fixed first order trend in the row direction in 2011 (β_row_ with associated indicator variable, *x*_*r-*ijkl_, indexing the row position in the field), and a fixed second order trend in the column direction in 2011 (β_col_ with associated indicator variable, *x*^*2*^_*c*_*-*_ijkl_, indexing the column position in the field). Of the 2480 inbred lines with available genotypic data, least squares means were estimated for 1687 lines from the combined model (File S4 in Additional file 1). Means were not estimable for the remaining lines due to missing phenotypic observations in all years (typically due to extreme time to maturity or poor seed production). Given the imbalance in the number of experimental entries in 2010 versus 2011/2012, a second filtered least squares mean data set was created that included only the 734 inbred lines for which we had data from all three years of testing (File S4 in Additional file 1).

Ear rot data from the 2011/2012 topcross experiments were analyzed using the same model selection protocol as the inbred experiments. The only difference in model selection in the topcross experiments was the testing of random set, group within set, and block within group effects in addition to other fixed and random effects tested in the inbred models. The combined model for the topcross experiments consisted of a fixed entry effect, random year and entry × year effects, a heterogeneous error variance structure within each year, and the significant spatial and experimental design factors nested within years: a fixed flowering time covariate in both years, an anisotropic correlated error structure in the row direction in both years, and a fixed first order trend in the row direction in 2011. From the combined model, least squares means were estimated for all 556 topcross hybrid entries. Means were then divided into two separate data sets based on tester. The B47 topcross set contained 243 means, and the PHZ51 topcross set contained 313 means (File S4 in Additional file 1).

Heritability of Fusarium ear rot resistance was estimated within the inbred association panel and topcross hybrid experiments. The same models used to estimate least square means were used to estimate heritability except entries were treated as random effects to obtain estimates of genetic variance. Entry mean-basis heritability was estimated as

$$ {\hat{H}}_C=1-\frac{\sigma_{PPE}^2}{2{\hat{\sigma}}_G^2} $$

where ${\sigma}_{PPE}^2$ is the average prediction error variance for all pairwise comparisons of entries and ${\hat{\sigma}}_G^2$ is the estimated genetic variance [42]. Five entry mean-basis heritabilities were estimated: across the full inbred association panel, within the filtered inbred subset of 734 lines, across all topcross hybrids, within the B47 topcrosses, and within the PHZ51 topcrosses.

Genotypic correlations between inbred rot resistance and hybrid rot resistance were estimated using individual location least square means for inbred entries and their corresponding topcross hybrids in a multivariate mixed model in ASReml. The least squares means used to calculate genetic correlations were only from years in which both inbred entries and hybrids were evaluated simultaneously (2011 and 2012). The model statement in ASReml was specified as

$$ {Y}_{INB},{Y}_{B47},{Y}_{PHZ51}= Trait+ Trait. Year+ Trait. Entry $$

where *Y*_*INB*_ is the inbred *per se* rot score variate, *Y*_*B*47_ is the B47 topcross hybrid rot score variate, *Y*_*PHZ*51_ is PHZ51 topcross hybrid rot score variate, *Trait* fits the mean for all three disease variates, *Trait.Year* fits a fixed year effect for each disease variate, and *Trait.Entry* fits the random genotype effect for each disease variate. Each term in the model was associated with one variance component for each trait and three covariance components between the three traits.

#### Association analyses

A genetic kinship matrix (**K**; File S5 in Additional file 1) for all 2480 inbred lines based on observed allele frequencies ([43]; method 1) was created using R software version 3.0.1 [44]. A subset of 10,241 SNP markers from the entire genotypic data set of 2480 inbred lines was used to produce **K**. The subset of markers was created by selecting markers from the complete marker set with less than 1% missing data. Missing genotypes remaining in the marker subset were imputed using a stochastic approach described by Zapata-Valenzuela et al. [45]. This method imputes a categorical genotype based on the frequency of all genotypes observed at the same locus across all individuals. This method imputes genotypic values that are expected to maintain the genotypic frequencies observed across the non-missing data. A principal components analysis in R was used to obtain the first two principal components of **K** in order to study the association of population structure with mean Fusarium ear rot scores.

The R package GAPIT version 3.35 [46] was used for the genome-wide association analyses based on a compressed mixed linear model [47]. Analyses were conducted on four sets of means: the entire set of inbred lines with phenotype data (1687 entries); the filtered set of inbred lines tested in all years (734 entries); the B47 topcross set (243 entries); and the PHZ51 topcross set (313 entries). In each set of means, missing values were included to allow for the same kinship matrix to be used across all analyses. The mixed linear model implemented by GAPIT was

$$ \mathrm{y}=\mathrm{X}\upbeta +\mathrm{Z}\mathrm{u}+\mathrm{e} $$

where **y** is the vector of ear rot least squares means on the natural-log scale, **β** is a vector of fixed effects including SNP marker effects, **u** is a vector of random additive genetic effects from background QTL for lines, **X** and **Z** are design matrices, and **e** is a vector of random residuals. The variance of the **u** vector was modeled as

$$ \mathrm{V}\mathrm{a}\mathrm{r}\left(\mathrm{u}\right)={\mathrm{K}\upsigma}_{\mathrm{a}}^2 $$

where **K** is the 2480 × 2480 matrix of pairwise kinship coefficients and ${\upsigma}_{\mathrm{a}}^2$ is the estimated additive genetic variance [47]. The full **K** matrix was used for all analyses.

Restricted maximum likelihood estimates of variance components were obtained using the optimum compression level and population parameters previously determined (P3D) options in GAPIT [47]. The positive false discovery rate (FDR) across all 200,978 tests of association between one SNP and ear rot resistance was estimated by GAPIT using the Benjamini - Hochberg method [48]. The MaizeGDB genome browser [49] was used to identify predicted genes either containing or located within 0.5 Mb of significant SNP hits from the GWAS. Annotations of predicted genes were combined from the maize reference sequence 5b filtered gene set (available from MaizeGDB; http://ftp.maizegdb.org/MaizeGDB/FTP/B73_RefGen_v2_dumps/) and the 6a reference sequence available at Phytozome V10 (http://phytozome.jgi.doe.gov/pz/portal.html) [31]. SNP positions were also converted to RefGen V3 positions to permit use of the Ensembl variant effect predictor tool (http://plants.ensembl.org/Zea_mays/Info/Index) to determine the type of mutation caused by SNPs [50].

The 1687 lines of the full inbred panel with phenotype data were grouped into one of five major maize subpopulations (stiff stalk, non-stiff stalk, tropical, popcorn, and sweet corn) based on pedigree information compiled by Romay et al. ([26]; http://genomebiology.com/content/supplementary/gb-2013-14-6-r55-s1.xlsx). Pedigree descriptors of the additional North Carolina State University lines added to the experiment in 2011 were obtained from http://www.cropsci.ncsu.edu/maize/germplasm.html and appended to the Romay et al. [26] data set. Lines of mixed ancestry (“unclassified”) were dropped from the analysis. Landraces were also dropped due to very small sample size. The frequencies of alleles that reduced disease severity at significantly associated SNPs from the GWAS were estimated within each subpopulation in R software, and a Fisher’s exact test was used to test the null hypothesis that the frequency of the allele conferring increased disease resistance was the same across all five subpopulations.

#### Data resampling analysis

To measure the robustness of GWAS associations detected in the full inbred panel analysis, we generated 50 subsample data sets, each containing phenotypic data from a random sample of about 80% of the inbred lines. Subsample data sets were generated in 10 replications in each replication the complete data set was partitioned into five folds, each fold containing an approximately equally sized random sample of lines. GWAS was conducted on each of the 50 subsample data sets in the same manner as for the full data set. The resample model inclusion probability (RMIP; [51]) for each SNP was computed as the frequency across the 50 data subsamples with which the SNP’s association test had a *p*-value less than 10^−5^.

### Availability of supporting data

The data sets supporting the results of this article are available at the Panzea.org repository: http://www.panzea.org/db/gateway?file_id=Zila_etal_2014_data_and_supp.

## Additional file

Additional file 1:
**Contains Figures S1 to S5, Table S1, and descriptions of supporting data Files S1 to S7.**

## Abbreviations

exPVPA: Expired plant variety protection act
FDR: False discovery rate
GBS: Genotype-by-sequencing
GWAS: Genome-wide association study
LD: Linkage disequilibrium
MAF: Minor allele frequency
NCRPIS: North central regional plant introduction station
NSS: Non-stiff stalk temperate
PC: Popcorn
QTL: Quantitative trait loci
SC: Sweet corn
SNP: Single nucleotide polymorphism
SS: Stiff stalk temperate
TS: Tropical/subtropical
USDA: United States Department of Agriculture
