# Supplementary material for: Genome-wide association study of Fusarium ear rot disease in the U.S.A. maize inbred line collection
Source: BMC Plant Biol. 2014 Dec 30;14:372. doi: 10.1186/s12870-014-0372-6 (PMC4302614; doi:10.1186/s12870-014-0372-6)
Supplement: Additional file 1: — Contains Figures S1 to S5, Table S1, and descriptions of supporting data Files S1 to S7. [file 12870_2014_372_MOESM1_ESM.docx]

**Genome-Wide Association Study of Fusarium Ear Rot Disease in the U.S.A. Maize Inbred Line Collection**

**ADDITIONAL INFORMATION**

Charles T. Zila^*^, Funda Ogut^*^, Maria C. Romay^†^, Candice A. Gardner^‡^, Edward S. Buckler^†,§,**,^, and James B. Holland^*,††1^

^*^Department of Crop Science, North Carolina State University, Raleigh, North Carolina 27695

^†^Institute for Genomic Diversity, Biotechnology bldg., Cornell University, Ithaca, NY, 14853, USA

^‡^U.S. Department of Agriculture—Agricultural Research Service, North Central Regional Plant Introduction Station, Ames, IA, 50014, USA

^§^U.S. Department of Agriculture—Agricultural Research Service, Plant, Soil, and Nutrition Research Unit, Ithaca, NY, 14853, USA

^**^Department of Plant Breeding and Genetics, Bradfield Hall, Cornell University, Ithaca, NY, 14853, USA

^††^U.S. Department of Agriculture—Agricultural Research Service, Plant Science Research Unit, Raleigh, North Carolina, 27695

^1^Corresponding author: USDA-ARS and Department of Crop Science, Campus Box 7620, Raleigh, NC, 27695-7616. Phone: (919) 513-4198. E-mail: james_holland@ncsu.edu.

**Files S1-S7**

**Supporting data**

Available for download at: http://www.panzea.org/lit/publication.html#2014

**File S1.** Raw phenotypic data from the inbred association panel experiments in 2010-2012, formatted for spatial analysis in ASReml software. Columns in the data file are as follows from left to right: year (1=2010, 2=2011, 3=2012), row (field position of plot from front of field to the back), column (field position of plot from left to right), set, block, plot, inbred names (Material), anthesis date (DTA, converted to the number of days after planting until anthesis), silking date (DTS, converted to the number of days after planting until silking), Fusarium ear rot score (rot_AVG, averaged across ears within the plot), number of ears scored within each plot (earno), the natural log transformation of the average ear rot score (logrot), first through fourth order polynomial row trend effects (R1-R4), and first through fourth order polynomial column trend effects (C1-C4). Observations with the “MISSING” qualifier in the Material column are placeholders for the purposes of spatial analysis in ASReml.

**File S2.** Raw phenotypic data from the topcross experiments in 2011 and 2012, formatted for spatial analysis in ASReml software. Columns in the data file are as follows from left to right: year (1=2011, 2=2012), row (field position of plot from front of field to the back), column (field position of plot from left to right), set, group, block, tester (1=PHZ51, 2=B47, 3=placeholder for check Pioneer 31G66, 4=placeholder for check NC478×GE440), maturity (1=early, 2=late), plot, hybrid names (Pedigree), inbred association panel parent of hybrid (Parent), anthesis date (DTA, converted to the number of days after planting until anthesis), silking date (DTS, converted to the number of days after planting until silking), Fusarium ear rot score (rot_AVG, averaged across ears within the plot), number of ears scored within each plot (earno), the natural log transformation of the average ear rot score (logrot), first through fourth order polynomial row trend effects (R1-R4), and first through fourth order polynomial column trend effects (C1-C4). Observations with the “MISSING” qualifier in the Material column are placeholders for the purposes of spatial analysis in ASReml.

**File S3.** Genotypic data in HapMap format consisting of 200,978 SNP markers on the 2480 genotyped entries from across the inbred and hybrid experiments, compressed in .zip format due to size. Data have been filtered to remove SNPs with greater than 20% missing data and minor allele frequencies less than 0.05.

**File S4.** Fusarium ear rot least square means for all 2480 genotyped entries from across the inbred and hybrid experiments, formatted for analysis in the R software package GAPIT. The Material column contains the line names, and the other columns are as follows: least square means for the full inbred association panel (Inbred_full), means for the filtered inbred association panel (Inbred_filt), means for the B47 topcrosses (B47), and means for the PHZ51 topcrosses (PHZ51). Missing means are denoted by the “NA” qualifier.

**File S5.** A 2480×2480 genetic kinship matrix (**K**) based on VanRaden (2008), formatted for analysis in the R software package GAPIT. The first column contains line names, and all other columns contain the pair-wise kinship coefficients between lines.

**File S6.** SNPs detected as significant at *p* < 10^-5^ within each of 50 data subsamples. The complete set of 1687 inbred lines was sampled using five-fold sampling scheme in which random but disjoint sets of approximately 20% of the lines were dropped from each fold (“cv”). This process was replicated (“rep”) ten times to generate 50 data subsamples of 80% of the lines each. GWAS was performed on each subsample and the effects of SNPs detected at *p* < 10^-5^ in each data sample are recorded in this file.

**File S7.** Predicted genes from maize B73 reference sequence 5a and 6b filtered gene sets within 0.5 Mb of significant associations. Annotation information from MaizeGDB and Phytozome10.


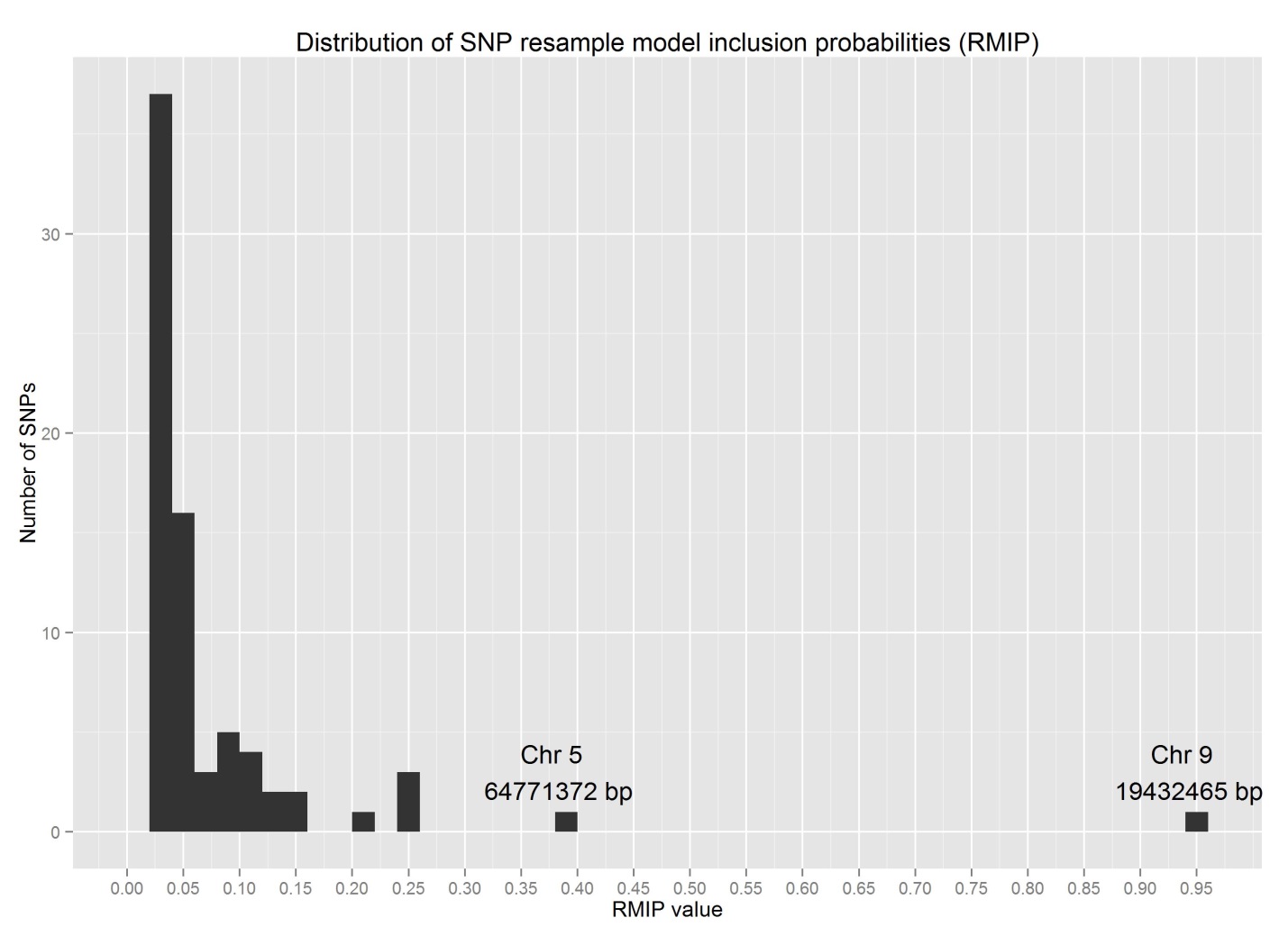


**Figure S1.** Distribution of resample model inclusion probabilities (RMIPs) for each SNP detected in at least one data subsample. Ten replicates of five folds each of the complete data set of 1687 inbred lines were sampled to generate 50 random data subsamples, each containing about 80% of the lines. GWAS was conducted within each subsample data set and the proportion of analyses in which a SNP was detected at *p* < 10^-5^ was recorded as the RMIP value for the SNP.

**
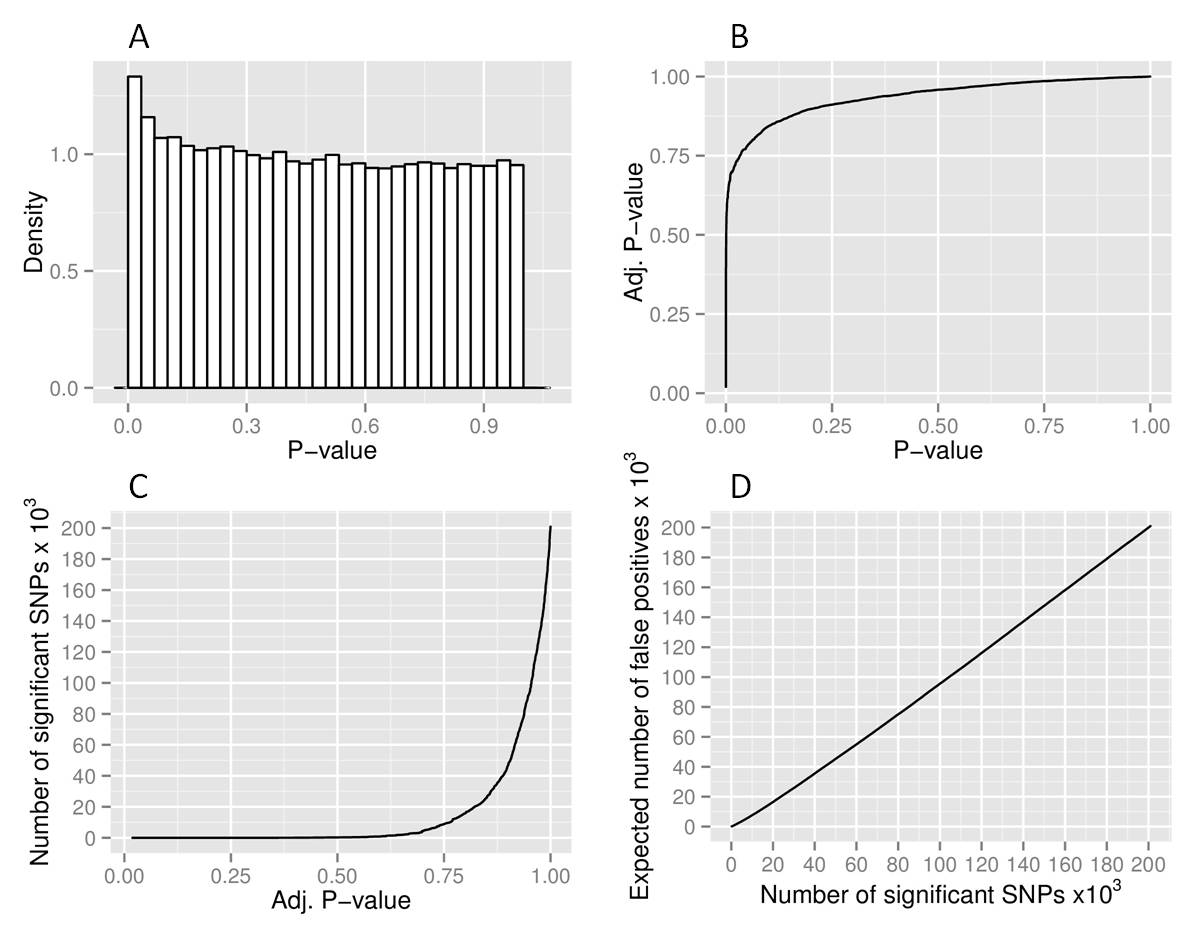
**

**Figure S2.** Estimating the false discovery rate (FDR) for SNP marker association with Fusarium ear rot resistance in the full inbred association panel analysis. (A) A density histogram showing raw *P*-value distribution of 200,978 SNPs following GWAS. (B) The FDR-adjusted *P*-values plotted against their respective raw *P*-values. (C) The number of SNPs plotted against each of the respective FDR-adjusted *P*-value estimates. (D) The expected number of false positive SNPs versus the total number of significant SNPs given the FDR-adjusted *P*-values.


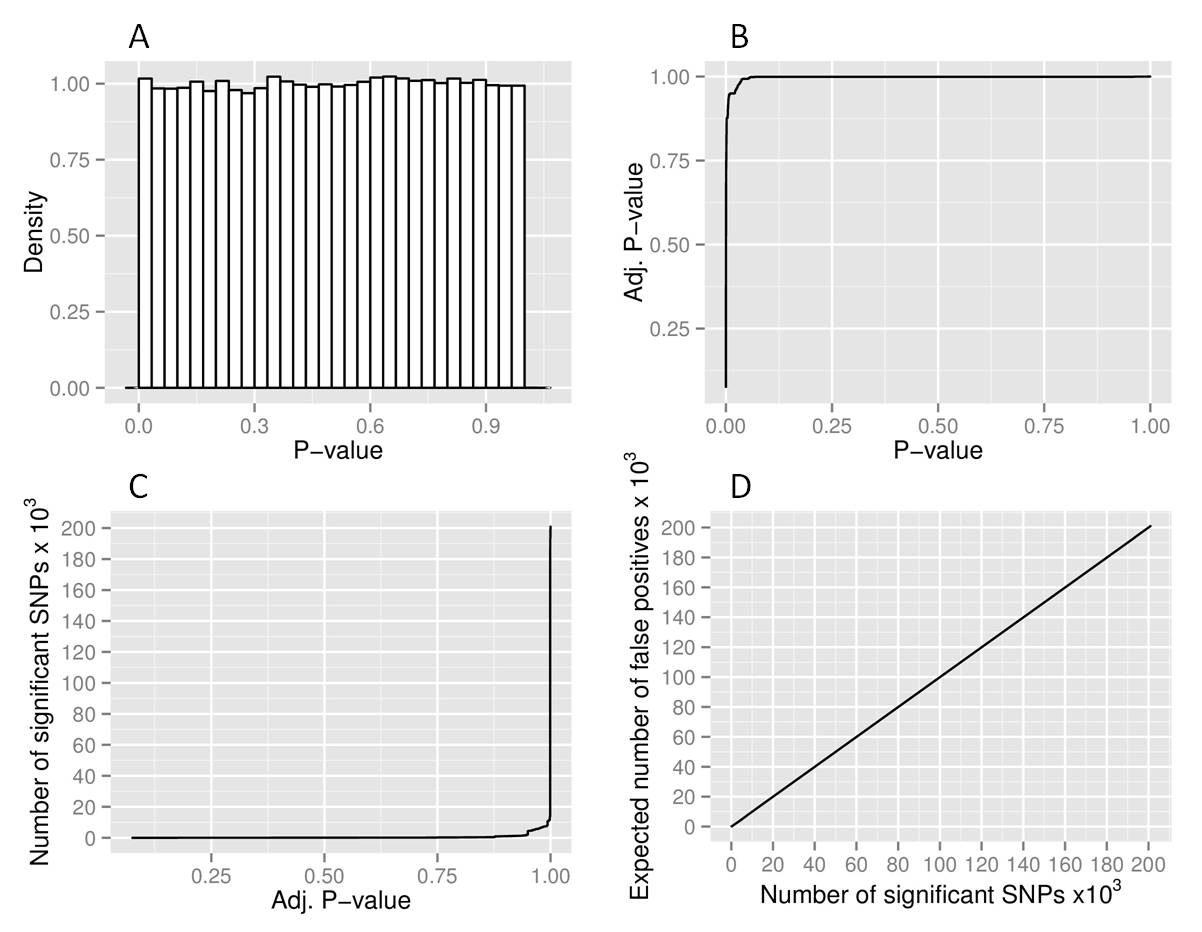


**Figure S3.** Estimating the false discovery rate (FDR) for SNP marker association with Fusarium ear rot resistance in the filtered inbred association panel analysis. (A) A density histogram showing raw *P*-value distribution of 200,978 SNPs following GWAS. (B) The FDR-adjusted *P*-values plotted against their respective raw *P*-values. (C) The number of SNPs plotted against each of the respective FDR-adjusted *P*-value estimates. (D) The expected number of false positive SNPs versus the total number of significant SNPs given the FDR-adjusted *P*-values.


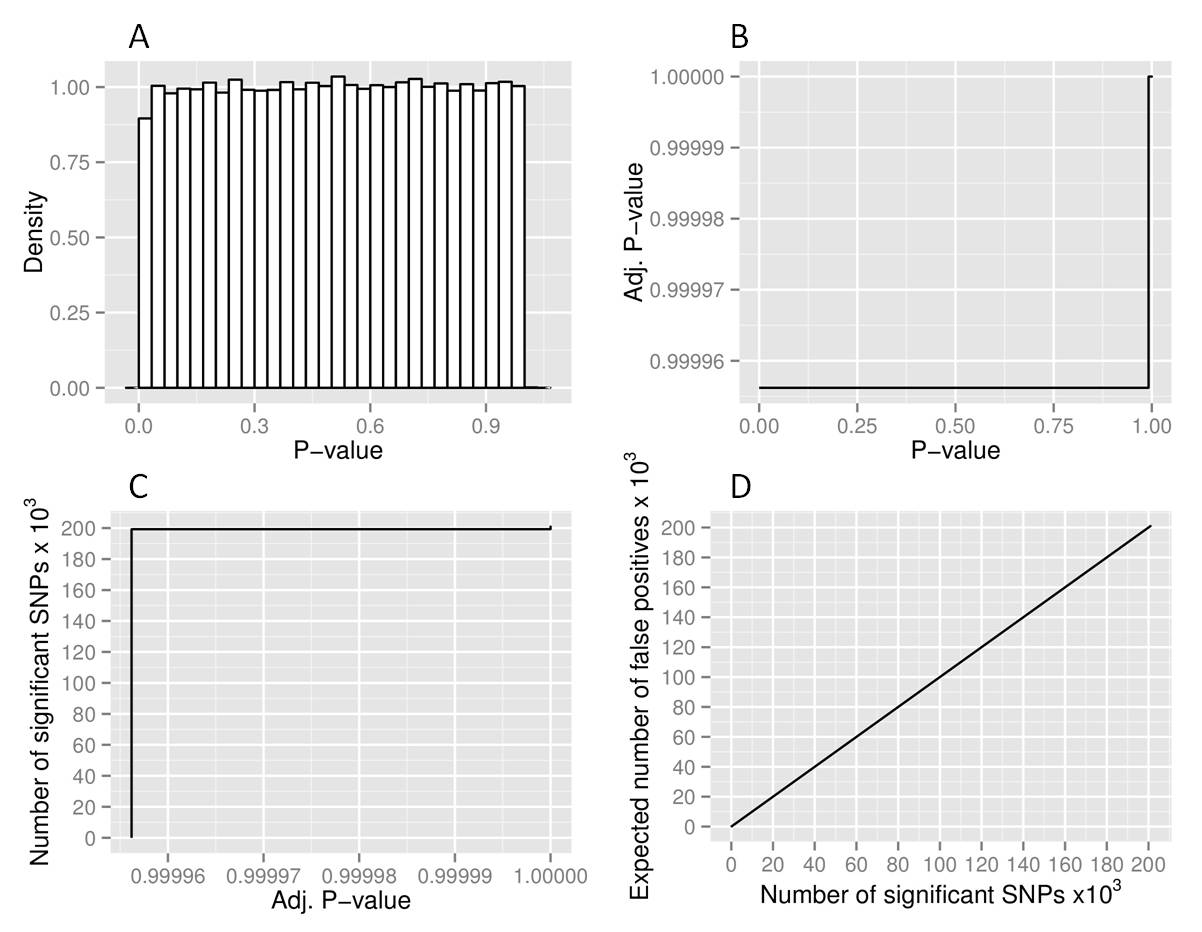


**Figure S4.** Estimating the false discovery rate (FDR) for SNP marker association with Fusarium ear rot resistance in the B47 topcross analysis. (A) A density histogram showing raw *P*-value distribution of 200,978 SNPs following GWAS. (B) The FDR-adjusted *P*-values plotted against their respective raw *P*-values. (C) The number of SNPs plotted against each of the respective FDR-adjusted *P*-value estimates. (D) The expected number of false positive SNPs versus the total number of significant SNPs given the FDR-adjusted *P*-values.


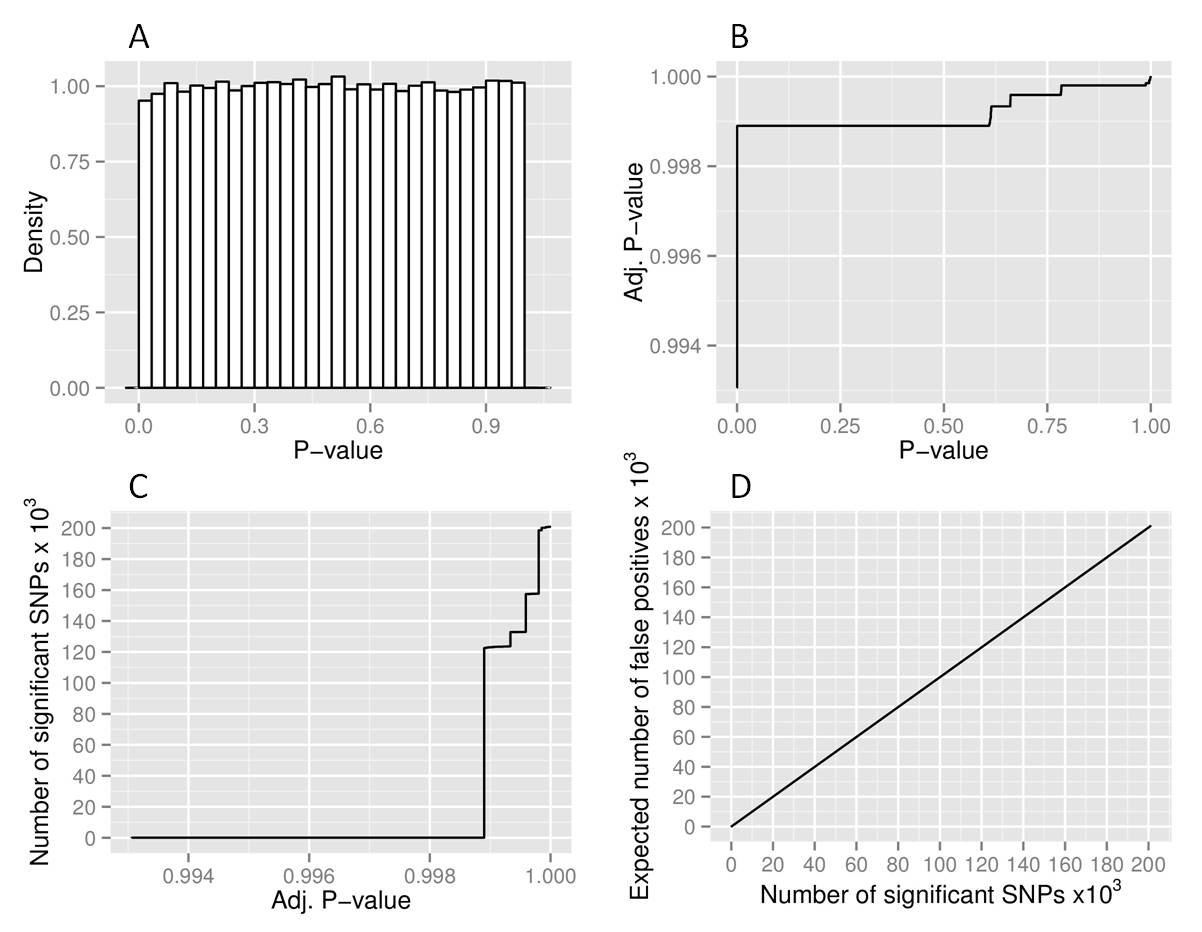


**Figure S5.** Estimating the false discovery rate (FDR) for SNP marker association with Fusarium ear rot resistance in the PHZ51 topcross analysis. (A) A density histogram showing raw *P*-value distribution of 200,978 SNPs following GWAS. (B) The FDR-adjusted *P*-values plotted against their respective raw *P*-values. (C) The number of SNPs plotted against each of the respective FDR-adjusted *P*-value estimates. (D) The expected number of false positive SNPs versus the total number of significant SNPs given the FDR-adjusted *P*-values.

Table S1. Comparison of GWAS results for seven selected SNPs between analysis in the full inbred panel (N = 1687) and the filtered balanced data set (N = 734).

| SNP |  | Minor allele frequency | |  | Minor allele effect estimate | |  | Raw p-value | |  | FDR adjusted p-value | |
| --- | --- | --- | --- | --- | --- | --- | --- | --- | --- | --- | --- | --- |
|  |  | Full | Filtered |  | Full | Filtered |  | Full | Filtered |  | Full | Filtered |
| S4_7566354 |  | 0.12 | 0.10 |  | -0.05 | -0.26 |  | 1.40E-01 | 7.34E-07 |  | 0.87 | 0.07 |
| S4_7618125 |  | 0.12 | 0.10 |  | -0.10 | -0.25 |  | 3.63E-03 | 2.67E-06 |  | 0.61 | 0.18 |
| S4_7618284 |  | 0.14 | 0.11 |  | -0.06 | -0.23 |  | 5.55E-02 | 3.96E-06 |  | 0.79 | 0.18 |
| S4_9353851 |  | 0.09 | 0.07 |  | -0.10 | -0.29 |  | 8.46E-03 | 6.14E-07 |  | 0.67 | 0.07 |
| S4_124930006 |  | 0.06 | 0.04 |  | -0.02 | -0.32 |  | 5.46E-01 | 4.36E-06 |  | 0.96 | 0.18 |
| S5_64771372 |  | 0.07 | 0.07 |  | -0.19 | -0.17 |  | 8.83E-07 | 1.10E-03 |  | 0.09 | 0.81 |
| S9_19532465 |  | 0.15 | 0.15 |  | -0.14 | -0.17 |  | 8.44E-08 | 2.15E-05 |  | 0.02 | 0.33 |
